# Nasolabial cyst: case report and review of management options

**DOI:** 10.1186/s12893-020-0677-3

**Published:** 2020-01-10

**Authors:** Abdulhakeem Almutairi, Abeer Alaglan, Mazyad Alenezi, Sultan Alanazy, Osama Al-Wutayd

**Affiliations:** 1 Department of Otolaryngology, Head and Neck Surgery, Qassim University, Buraydah, Saudi Arabia; 2 Qassim University, Buraydah, Saudi Arabia; 3 College of Medicine, Qassim University, P.O. Box 6655, Buraidah, Qassim 51452 Saudi Arabia; 4 Department of Family and Community Medicine, Unaizah College of Medicine, Qassim University, Buraydah, Saudi Arabia

**Keywords:** Nasolabial, Cyst, Maxillofacial cyst, Otorhinolaryngology

## Abstract

**Background:**

Nasolabial cysts are rare, non-odontogenic, soft-tissue cysts that develop between the upper lip and nasal vestibule with an overall incidence of 0.7% out of all maxillofacial cysts. The predominant presentation of a nasolabial cyst is a painless localized swelling with varying degrees of nasal obstruction. Several treatment modalities have described in the management of the nasolabial cyst. In this paper, we present a case of a nasolabial cyst in a 44 years old man with discussions of the treatment modalities in the lights of the literature.

**Case presentation:**

We present a case of a nasolabial cyst in a 44-year-old man that slowly increased in size through a period of 3 years, with associated mild pain and nasal obstruction. It had caused a mass effect upon the maxilla, resulting in scalloping. The cyst was excised entirely with no evidence of recurrence at the two months follow up.

**Conclusions:**

The nasolabial cyst is a rare soft-tissue cyst. Complete surgical excision using an open approach performed to our case, which considered with the complete endoscopic removal of the best treatment for the nasolabial cysts with a rare recurrence rate.

## Background

Nasolabial cysts are rare soft tissue non-odontogenic cysts that develop between the nasal vestibule and upper lip [1]. The incidence of nasolabial cysts is 0.7% of all maxillofacial cysts. The size measures 1 to 5 cm in diameter [2]. These cysts in 90% of cases are unilateral, and 10% bilateral, they are commonly seen in the black women in the fourth to fifth decades of life [3]. Zuckerkandl was the first to describe the cyst in 1882. It is not uncommon to misdiagnose nasolabial cysts and not treat them appropriately because of their rarity [4]. The pathogenesis is uncertain with multiple theories. In 1920 Bruggemann proposed the most acceptable theory, which suggests that the nasolabial cyst arises from the remnants of the epithelium in the anterior lower part of the nasolacrimal duct [5]. The origin of the cyst is developmental, although it does not manifest until adulthood, and the typical presentation of a nasolabial cyst is a painless localized swelling with varying degrees of nasal obstruction [6]. The location and presentation of these cysts make them diagnosis nearly clinical exclusively. The diagnosis tests include nasal scope, CT and MRI. Both CT and MRI are valuable in revealing the origin of the cysts and avoids unnecessary needle aspiration or dental surgery [7, 8]. Surgery is equally diagnostic and curative by allowing histological examination [9]. In this paper, we present a case of a nasolabial cyst in a 44 years old man.

### Case presentation

A 44 years old medically free male began to complain of a right nasal swelling three years ago. It has fluctuated in size in the previous three years. Recently, it started to slowly increase in size with associated mild pain and nasal obstruction. The patient denied any history of medical disease, history of trauma or surgery.

**Fig. 1:**
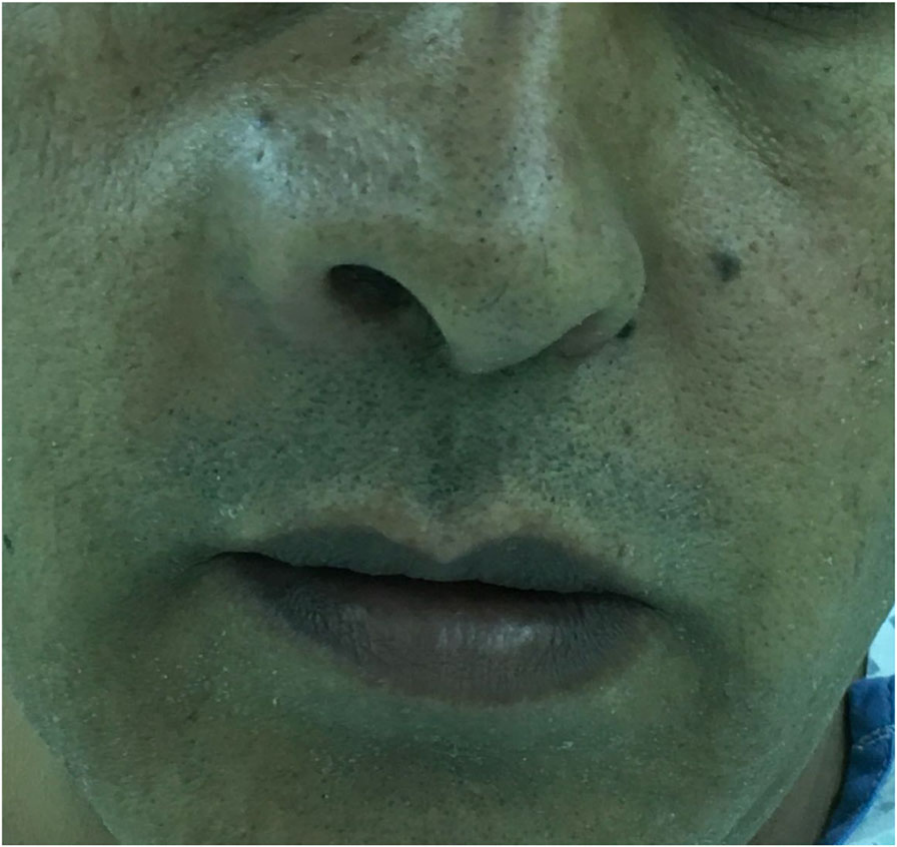
Upon initial examination, the patient had a mass in the right nasolabial area with associated facial asymmetry. There was no discharge or overlying skin change

On examination: There was a right nasolabial mass, 3 × 4 cm, round fluctuating, no discharge, or overlying skin change (Fig. 1).

**Fig. 2:**
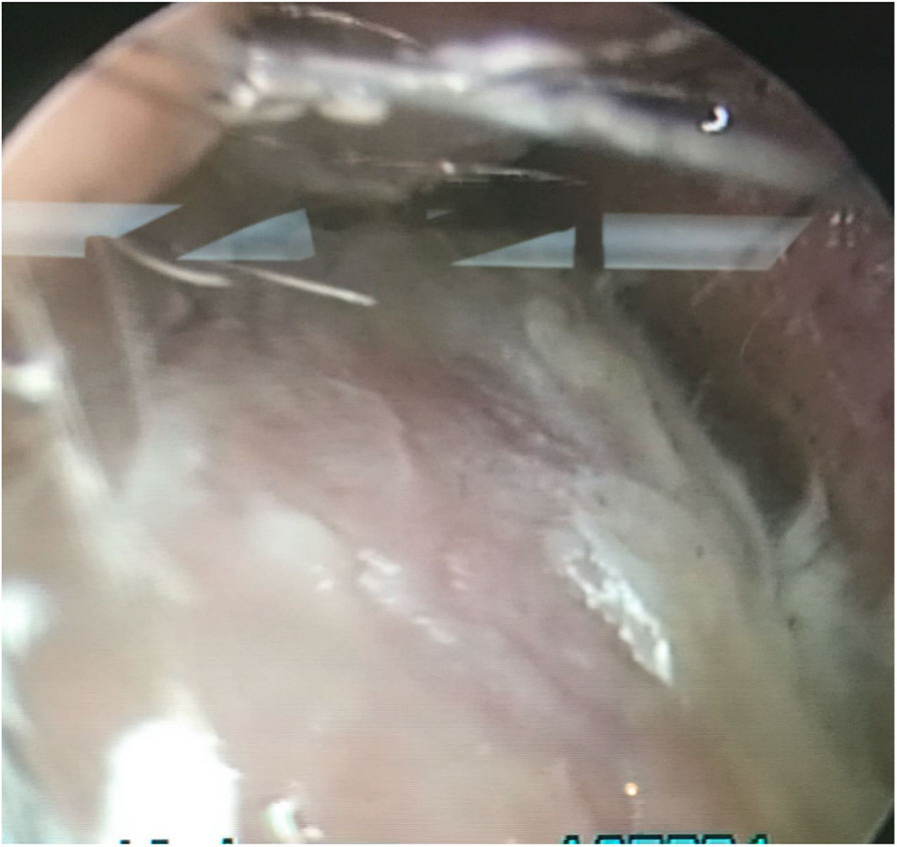
Endoscopy revealed a mass obstructing most of the right nasal aperture

There was mild tenderness on palpation. The endoscopic examination showed a mass obstructing most of the right nasal aperture (Fig. 2).

**Fig. 3:**
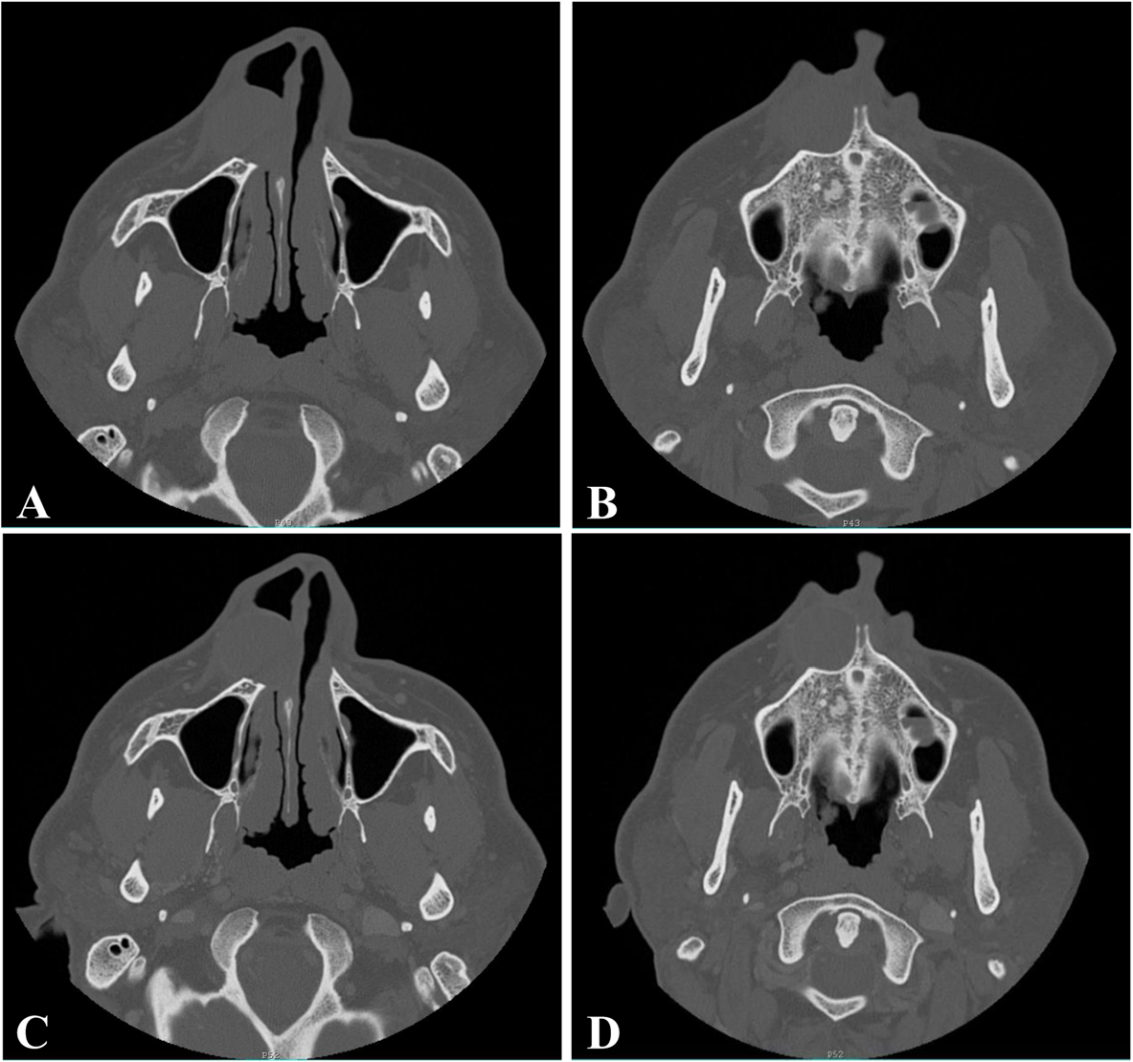
**a**, **b**: CT without contrast showing a right inferior nasal alar region mass measuring 3.2*2.2*2.5 cm exhibiting an isodense to hypodense texture. There is a mass effect upon the maxilla causing scalloping. No bone destruction. **c**, **d**: CT with contrast showing no significant enhancement within the mass

CT scan done showed a right inferior nasal alar region space-occupying lesion, measuring 3.2 × 2.2 × 2.5cm, which exhibits isodense to hypodense texture. There was no enhancement or bone destruction. It was causing a mass effect upon the maxilla, causing scalloping (Fig. 3).

**Fig. 4:**
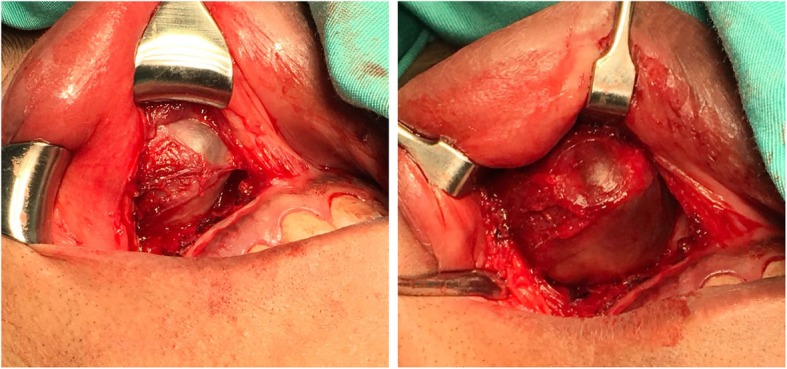
Intraoral approach to excise the nasolabial cyst through a sublabial incision in the upper buccal sulcus

A final diagnosis of unilateral nasolabial cysts was given based on the clinical and CT scan finding. So, no further workup. The cyst excised by the sublabial approach (Fig. 4).

**Fig. 5:**
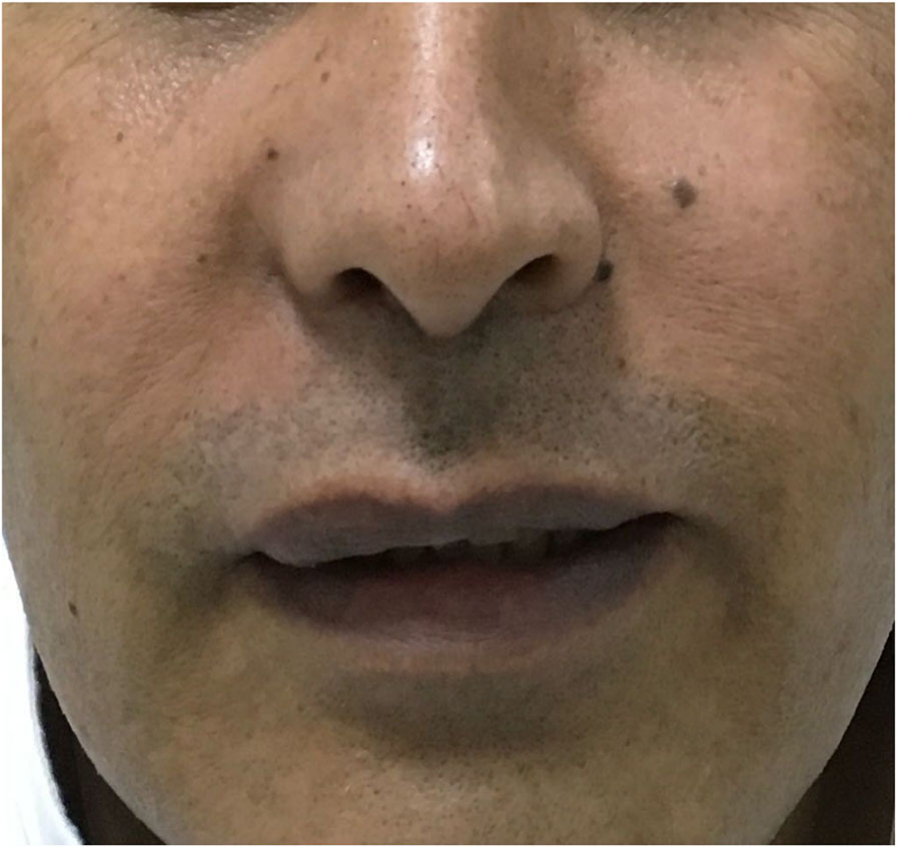
Two months postoperatively, there is no facial edema and no signs of cyst recurrence

They were starting by upper gingivolabial sulcus incision below the piriform apertures. A round, smooth, extraosseous cystic swelling superficial to the anterior wall of maxilla. The cysts were excised entirely with no attachment to the underlying bone and were firmly adherent to the floor of the nasal cavity in the mucocutaneous junction region. Part of the skin and mucosa removed in the right nasal cavity as it was part of the cyst wall. Dead space was packed by iodinated gauze and removed from the nose after 24 h. The wound closed by 3–0 Vicryl. The histopathological examination showed respiratory epithelium (ciliated pseudostratified columnar) with goblet cells compatible with nasolabial cysts. Postoperatively, the patient had mild facial edema with numbness over the right upper lip and teeth. He was seen after two weeks to remove stitches and intranasal cavity wound healed well. Edema had subsided by then, while the patient still reported numbness in the right upper lip and teeth. Two months after surgery, he has seen with an improvement of the previously reported numbness and fourteen months follow up showed no recurrence of the mass (Fig. 5).

There was no indication for radiological examination as there were no complications or recurrence of the lesion.

## Discussion and conclusions

The nasolabial cyst is a rare condition and accounts for about 0.7% of all cases of maxillofacial cysts, and only 2.5% of the maxillofacial non-odontogenic cysts [4]. It’s believed that its occurrence is more than that reported in the literature; though, indexes are limited owing to high rates of misdiagnosis [6]. These cysts are unilateral in 90% of cases and 10% bilateral. Unlike our case, they are seen commonly in black women in the 4th to 5th decades of life [3]. Their extra-osseous origin and location recognize nasolabial cysts under the alae nasi [10]. It has been given multiple names since, including nasoalveolar and Klestadt cyst. Later, in 1951, the term nasolabial cyst was introduced by Rao [10]. This term has been regarded as more accurate since the cysts are situated wholly within soft tissue, unlike nasoalveolar cysts, which typically cause a maxillary bone defect [3, 8]. It has recognized because of the complex discussions of its pathogenesis, as well as its characteristic clinical presentation [11]. Bruggemann proposed the most acceptable theory in 1920, which suggests that the nasolabial cyst arises from the epithelial remnants of the lower anterior part of the nasolacrimal duct [5]. Although the cyst is developmental in origin, it typically does not manifest until adulthood, which was the case in our patient [6]. Characteristically, a patient would present with painless swelling, mostly in the left side of the upper lip adjacent to the nasal alae, and very slow-growing nature [12]. These cysts vary in size from 1 cm to 5 cm, and infrequently erode the underlying bone if they grow to a large size [8]. The submucosal location of nasolabial cysts at the anterior nasal floor is both distinctive and constant. It was described by Bull et al. in 1967 as essentially pathognomonic [13]. Arising from this location, the growth of the cysts can be possible in three directions: to the nasolabial fold, the mouth vestibule, and the nasal vestibule [13]. Patients with nasolabial cysts can be asymptomatic; however, most have at least one of the three key symptoms: partial or complete nasal obstruction, well-circumscribed swelling, or localized pain [4]. Each of the key symptoms found in our patient. The signs of the cysts are rather specific [7]. Comprising of a fluctuant swelling of the maxillary labial fold and the floor of the nasal vestibule, obliteration of the nasolabial fold and elevation of the nasal alae [6, 7]. A well-localized fluctuating swelling with a cystic consistency in the nasolabial sulcus has been reported as a definitive sign of a nasolabial cyst by Graamans et al. [8]. In nearly 30% of patients, the initial presentation is an infection. In one series done by Kuriloff, half of the patients developed an infection [13]. Once infected, the cyst becomes painful and could rupture spontaneously to drain into the oral cavity or nose [14]. The presentation of nasolabial cysts is variable, patients treated by several practitioners, including plastic surgeons, otolaryngologists, and others [13]. The differential diagnosis of nasolabial cysts is made straightforward by their extraosseous location. The dentoalveolar abscess is the most relevant differential, which can be excluded easily by testing the affected teeth vitality [4, 14]. It also includes the oronasal cysts, especially the commonest non-odontogenic maxillary cystic lesion, nasopalatine duct cyst [8]. Since, the nasolabial cyst is an extraosseous soft tissue mass, and it can easily differentiate from the nasopalatine duct cyst with the help of MRIs as the latter is an intraosseous cyst [15]. The nasolabial cyst should be differentiated from dermoid and epidermoid cysts, as the color of the mucosa is yellow discoloration, while in nasolabial cysts, the color of the mucosa is natural pink hue or like blue-tinged. Additionally, epidermoid and dermoid cysts are typically in childhood, whereas nasolabial cysts more commonly seen in an adult [7]. Other differential diagnoses include sebaceous cysts, as well as malignant or benign salivary gland tumors [4]. To a lesser extent, the infection spread from the cysts can mimic acute maxillary sinusitis, periodontal abscess, nasal furunculosis, or facial cellulitis [13]. The tests for diagnosis include nasal scope, CT and MRI [8]. Both CT and MRI are valuable in revealing the origin of the cysts and avoids unwarranted needle aspiration or dental surgery [3, 7].

Ultrasonography could be used in an office-based diagnostic tool for the nasolabial cyst [16]. Diagnostic CT scan is of high significance and relatively low cost. It described as the imaging modality of choice for evaluation of the lesion borders is required. Therefore, CT is considered essential for the preoperative estimation of lesion extent and limitation [17]. Cysts located classically anteriorly to the piriform aperture [13]. However, a definite diagnosis can reach through histological examination [4, 7, 13]. Hence, resection of the cyst is both diagnostic and curative by allowing histological examination [9]. Several modalities in the nasolabial cysts management include endoscopic marsupialization, surgical excision, incision and drainage, injection of sclerotic agents, simple aspiration and cauterization. Excluding endoscopic marsupialization and complete surgical excision, all the other modalities have a high recurrence rate [8, 13]. Sheikh et al. reviewed 79 articles with 311 patients with a nasolabial cyst and reported no significant recurrence rate found between the sublabial and transnasal marsupialization excision [18, 19]. Nearly in all published literature, complete surgical excision described as the best treatment for the nasolabial cyst [6, 13]. It is successful with rare recurrence of the cyst [20]. The indications of surgery are, to establish a diagnosis, prevent infection of the cyst, and to improve any cosmetic deformity [13]. The commonest implemented approach is intraoral enucleation through local anesthesia by a sublabial incision in the upper buccal sulcus, which allows a surgical field to be wider and more guarantee of an excision completely without tearing the nasal mucosa or entering the maxillary sinus [5, 8, 9, 13]. As nasolabial cysts situated near the nasal cavity floor, perforation of the mucosa during excision can happen. This complication is not uncommon, and once it occurs, should be closed with sutures to avoid oronasal fistula formation [7]. Some authors advocate that when small perforations caused, they can be left untreated with gentle vestibule packing; however, they must be sutured the larger one [6, 13]. They are other complications like wound infection, soft-tissue swelling, and hematoma [21]. In 1999, an alternative described by Su et alin the means of endoscopic trans-nasal marsupialization, which is effective and simple [5]. It is assumed to be an easier approach for large lesions. Using this approach is especially advantageous when the cyst extends to the floor of the nose, which would increase the perforation risk and defects with the conventional sublabial approach [22]. Endoscopic marsupialization, when compared to the conventional incision, is rapid and can be done in an outpatient setting, with an operative time of approximately 15 min to complete each procedure [23, 24]. Additionally, intraoperative bleeding was minimal, and no postoperative pain or edema reported [24]. Since the nasolabial cyst lined with ciliated respiratory epithelium, it is converted by marsupialization into a sinus at the anterolateral nasal floor [22]. It is designed to be like a healthy paranasal sinus with good drainage and best ventilation functions, without subsequent mucus accumulation [23]. Nevertheless, if the window created during marsupialization is too small, it leads to shrinking the annular scar around the ostium and followed by accumulation of the mucus in the newly created sinus or cyst recurrence [23]. Hence, recurrence reported following this modality in recent reports [5, 17]. Another approach to surgically remove the nasolabial cyst is the Neumann incision [25, 26]. It is more commonly used by endodontists to perform alveoloplasties rather than excising nasolabial cysts [25]. Still, this approach takes into consideration the elaborate anatomy of nerves and blood vessels in the region; therefore, the disturbances can be local and seen with the previously described sublabial approach, such as bleeding and teeth numbness, are minimal [25]. The Neumann incision is particularly useful when dealing with a large cyst as a complete cyst excision and best access to the pyriform aperture [25, 26].

In conclusion, nasolabial cysts are rare soft-tissue cysts. It is believed that its occurrence is more than that reported in the literature. Complete surgical excision using an open approach done to the patient and allowed for histological examination and considered the best treatment for nasolabial cysts. Furthermore, excluding complete surgical removal and endoscopic marsupialization, all other modalities are associated with a high recurrence rate.

## Data Availability

All data generated or analyzed during this study included in this published article and its supplementary information files.

## Abbreviations

CT: Computed tomography
MRI: Magnetic resonance imaging
